# A Case of Chronic Deep Infrapatellar Bursitis Complicated by Patellar Tendinopathy and its Evaluation With Musculoskeletal Ultrasound

**DOI:** 10.7759/cureus.22057

**Published:** 2022-02-09

**Authors:** Debasish Jena, Apurba Barman, Jagannatha Sahoo, Ranjan Patel, Anchal Dalai

**Affiliations:** 1 Physical Medicine and Rehabilitation, All India Institute of Medical Sciences, Bhubaneswar, Bhubaneswar, IND; 2 Radiodiagnosis, All India Institute of Medical Sciences, Bhubaneswar, Bhubaneswar, IND; 3 Anaesthesiology, Srirama Chandra Bhanja Medical College and Hospital, Cuttack, IND

**Keywords:** musculoskeletal ultrasound, ultrasonography, knee pain, patellar tendinopathy, deep infrapatellar bursitis, bursitis

## Abstract

Knee pain is a very common complaint in routine physiatry and orthopedic practice. While bursitis is a well-known and common cause of knee pain, deep infrapatellar bursa (DIPB) involvement is relatively less common. Inflammation of DIPB occurs commonly due to either direct trauma or overuse, but other rare causes have also been reported in the literature including infection, juvenile idiopathic arthritis, gout, and juvenile ankylosing spondylitis. We report a case of chronic inflammation of DIPB caused by direct trauma and associated with patellar tendinopathy. Additionally, we describe the characteristic findings on musculoskeletal ultrasonography (MSK-USG). For ultrasound evaluation, the patient should lie supine with the knee slightly flexed. Deep infrapatellar bursitis can be seen as an anechoic fluid-filled structure immediately posterior to the distal patellar tendon and anterior to the tibial tuberosity. While MRI can confirm the diagnosis of bursitis, MSK-USG can be quick, highly sensitive, and is able to confirm the diagnosis as well as to detect dynamic changes in the patellar tendon and adjacent structures. USG can also help in the treatment by guiding corticosteroid injection into the bursa. Activity modification and eccentric exercises play an important role in the rehabilitation program in these cases.

## Introduction

Bursitis is defined as the inflammation of a bursa, which is a synovium-lined, fluid-filled, sac-like structure typically found around large joints such as the hip, knee, shoulder, and elbow. The major role of a bursa is to reduce the friction between adjacent moving structures such as skin and tendon or tendon and bone [1]. The common causes of bursitis are overuse, direct trauma or repetitive microtrauma, crystal disease, hemorrhage, infection, and systemic inflammation [2]. Although bursitis is quite common among the general population, pathology of the deep infrapatellar bursa (DIPB) is less commonly encountered in routine practice. DIPB is located between the posterior aspect of the distal patellar tendon and the anterior aspect of tibia tuberosity [3]. Due to the relatively deeper location of DIPB, it may become difficult to diagnose the condition with clinical examination alone. The role of MRI in the evaluation of bursitis has already been established but the role of ultrasonography (USG) is still not very clear. Also, the authors could not find any published literature that has demonstrated the association of patellar tendinopathy with chronic inflammation of DIPB by USG evaluation. In this report, we present a case of chronic inflammation of DIPB of traumatic etiology complicated by the presence of patellar tendinopathy and its evaluation with musculoskeletal USG (MSK-USG).

## Case presentation

**Figure 1:**
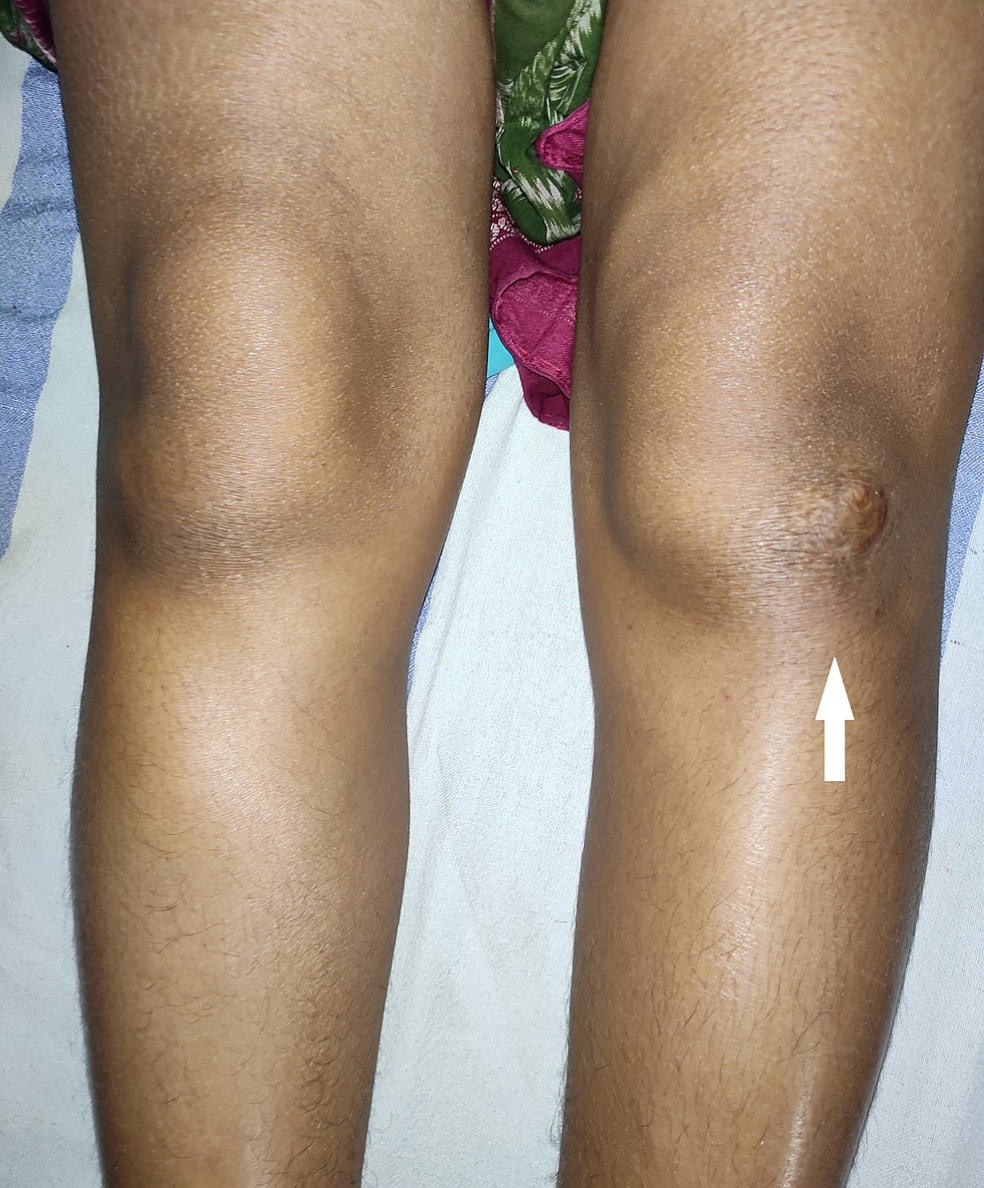
Clinical picture showing mild swelling and erythema at the inferolateral aspect of the patella on the left knee (white arrow)

A 32-year-old woman with a history of a fall from a bike one year prior presented with chronic anterior knee pain on the left side. The pain was intermittent, dull-aching, mild to moderate in severity, and present for the past 10 months. The average score of pain on the visual analog scale was 5/10. The patient was a housewife and she provided no history of any chronic medical illness, frequent kneeling, or jumping activities. She had already consulted many physicians for the intermittent pain exacerbations and was able to manage the pain with painkillers only without the condition being diagnosed. However, she had gradually developed persistent discomfort over the anterior knee and the pain was now aggravated by kneeling, heavy activities, and extreme knee flexion and extension. On careful inspection, there was mild swelling over the inferolateral aspect of the patella along with slight erythema over the area. Also, there was a healed scar mark adjacent to the painful area (Figure 1). On palpation, the painful area was slightly warm to touch with point tenderness of grade 2 just lateral to the distal patellar tendon. Additionally, there was grade 1 tenderness of the distal one-third of the patellar tendon. On the assessment of knee range of motion, the movement was painful at the extremes of knee flexion and extension. No other positive findings could be elicited from a thorough knee examination and a provisional diagnosis of deep infrapatellar bursitis was made.

**Figure 2:**
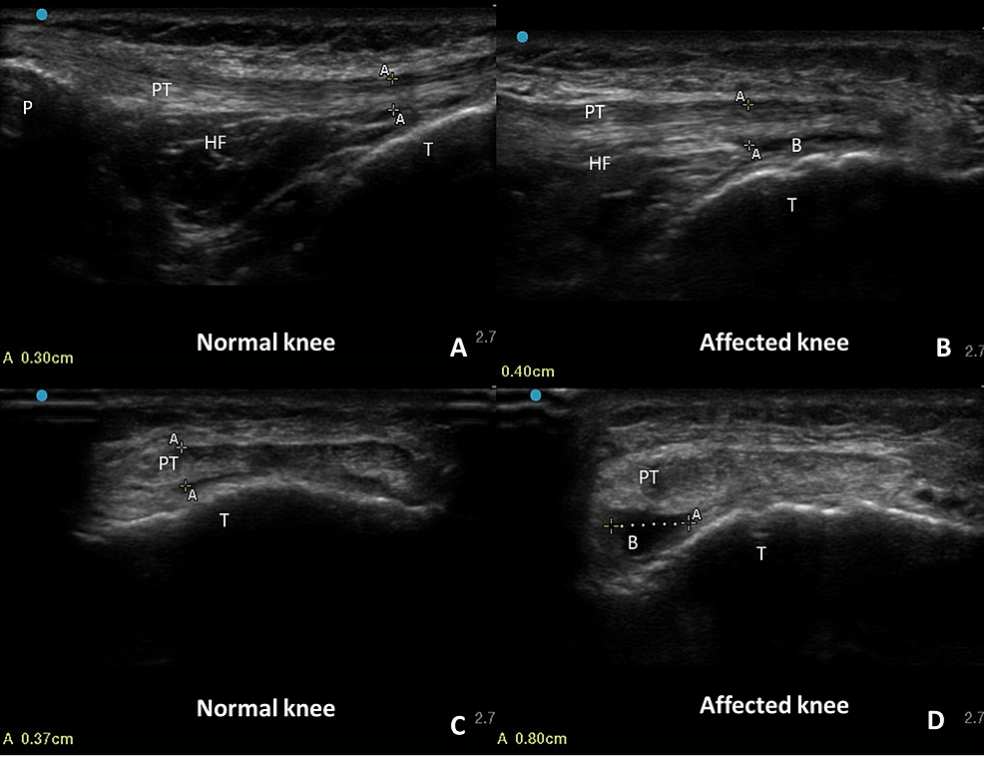
Longitudinal (A) and transverse scan (C) of the infrapatellar region of the healthy knee showing normal patellar tendon, Hoffa’s fat pad, and no appreciable bursa. Longitudinal (B) and transverse scan (D) of the infrapatellar region of the affected knee showing distended deep infrapatellar bursa, suggesting bursitis along with thickened and mildly heterogeneous patellar tendon, suggesting patellar tendinopathy P: patella; T: tibia; PT: patellar tendon; B: bursa; HF: Hoffa's fat pad

Routine blood investigations were within normal limits. A plain radiograph of the knee joint including anteroposterior, lateral, and skyline views was performed in a standing position, which revealed no abnormalities. Further, a USG evaluation was done with a linear probe of frequency 15-6 MHz (FUJIFILM Sonosite, Inc., Bothell, WA). The anterior knee evaluation was done with the patient lying supine and the knee in 20-30 degrees of flexion. The contralateral (healthy) knee was evaluated first, followed by scanning of the affected knee in both longitudinal and transverse planes. The affected knee showed an anechoic fluid-filled area immediately posterior to the distal patellar tendon and anterior to the tibia, suggesting a distended DIPB. The distended bursa was compressible under the probe with positive probe tenderness. In addition, the patellar tendon was thickened and showed a heterogeneous echotexture (Figures 2A-2D). Colour Doppler did not show any discernible increase in vascularity. Finally, a diagnosis of deep infrapatellar bursitis associated with patellar tendinopathy was made.

**Figure 3:**
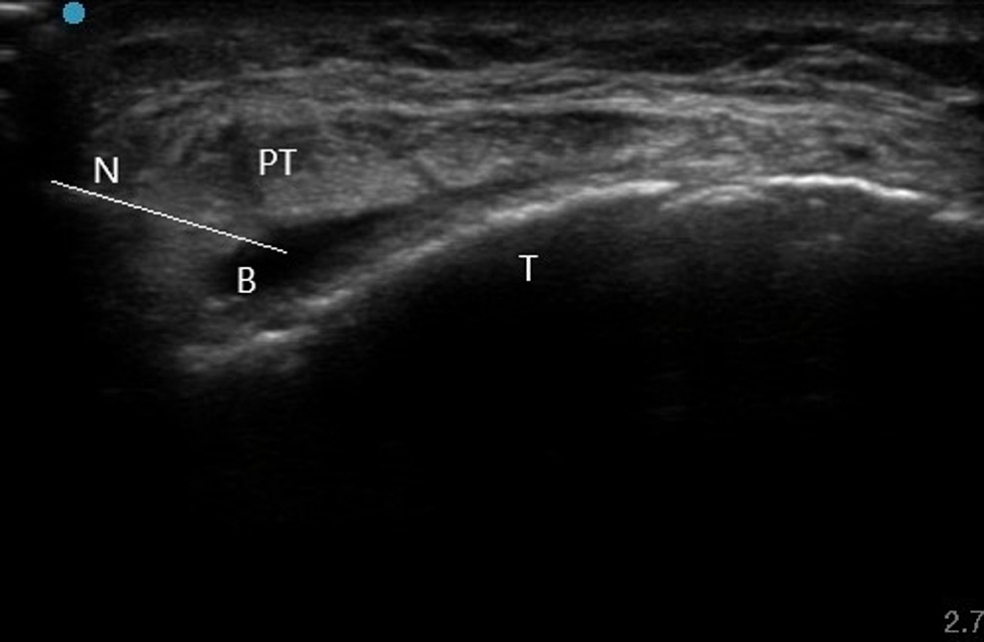
Injection of the deep infrapatellar bursa with an in-plane and lateral approach N: needle; PT: patellar tendon; B: bursa; T: tibia

USG-guided injection of 20-mg triamcinolone hexacetonide plus 2-ml lignocaine hydrochloride (total 2.5 ml) was instilled in the bursa in an in-plane and lateral approach with no post-procedural complications (Figure 3). In addition, the patient was advised local ice application thrice daily for 15 minutes each for a period of one week along with activity modifications. After one week of injection, quadriceps stretching exercises were started followed by eccentric strengthening exercises, two times a day, consisting of three sets of 15 repetitions each. The patient was followed up for three months and complete resolution of symptoms was observed with occasional activity-related discomfort in the anterior knee attributable to tendinopathy. She was advised to continue with eccentric quadriceps strengthening exercises and activity modifications.

## Discussion

Knee pain is very common among the general population and may remain undiagnosed for years. Deep infrapatellar bursitis is a relatively uncommon cause of knee pain as compared to the involvement of other common bursae including prepatellar, suprapatellar, and superficial infrapatellar bursa. Inflammation of the DIPB can be difficult to detect due to its location deep into the patellar tendon. Nonetheless, swelling may be seen adjacent to the distal patellar tendon and point tenderness can be elicited. It is frequently seen in runners and jumpers due to overuse of the extensor mechanism of the knee joint [4]. Deep infrapatellar bursitis has also been reported in the literature in association with several other conditions including infection, Osgood-Schlatter disease, gout, spondyloarthropathies, and juvenile idiopathic arthritis [4-8].

Routine laboratory investigations are usually performed to rule out other rare causes of bursitis including infection and systemic inflammation. Aspiration of the inflamed bursa may be helpful whenever there is a question of infection or bursitis secondary to crystalline disease. A plain radiograph may be done in suspected cases of traumatic etiology, Osgood-Schlatter disease, or associated calcification in the bursa. MRI is considered the imaging modality of choice to diagnose this condition. On MRI, inflamed DIPB appears as a triangular fluid collection immediately posterior to the distal patellar tendon [2]. However, because of the higher cost and delays in getting a scan in centers with high patient load, MRI seems to be less feasible. In such a situation, USG plays a pivotal role as it is quick, easily available, and cost-effective.

For sonographic visualization of DIPB, the patient should lie supine or in a long sitting position with the knee in 30 degrees of flexion. This slight flexion straightens the patellar tendon, thereby providing an overall better image of the DIPB [9]. Care should be taken not to apply any extra pressure with the transducer head on the area of scanning, as this can cause any small bursa to become flattened leading to its non-visualization. The examination should be done preferably in two perpendicular planes (longitudinal view and transverse view) and measurements of the bursa should be taken in both planes. The DIPB may be appreciated in healthy knees as a 2-3 mm, flattened, anechoic fluid-filled structure [9]. Similarly, the patellar tendon needs to be scanned along its length from the inferior border of the patella to its attachment over tibia tuberosity. In patellar tendinopathy, USG may reveal loss of anisotropy and an increase in tendon thickness with a heterogenous echotexture (with interspersed hypoechoic areas) in both planes. Colour Doppler may show increased flow indicating an ongoing inflammation [10]. Although increased flow was not observed on Colour Doppler in our case, the involved knee showed distended DIPB with a thickened and heterogeneous patellar tendon. Additionally, with real-time scanning, the pain may be reproduced during transducer palpation, further suggesting the presence of inflammation in the bursa and/or tendon. Most importantly, examination of the asymptomatic side is of great value to compare the findings before reaching any conclusions.

Initial management of DIPB should include relative rest, ice application, and anti-inflammatory medications. Activity modification may include avoidance of certain specific activities including jumping, running, and frequent kneeling. Aspiration of the bursa may be done in cases with significant distension, and routine microscopy and culture may be obtained in suspected cases of infection or crystal deposition disease. In recurrent or resistant cases of inflamed DIPB, an appropriate steroid injection is indicated. The use of USG guidance in either an in-plane or an out-of-plane approach can improve the accuracy of the procedure, thereby preventing intra-tendinous injection. In cases of associated patellar tendinopathy, stretching and eccentric exercises along with activity modifications should be incorporated in the rehabilitation program.

Besides the inherent limitations of a case report, our study is limited by the absence of an MRI of the patient. However, MSK-USG confirmed the diagnosis and clearly delineated the inflamed bursa as well as the patellar tendon thickening. Also, the causal association between chronic deep infrapatellar bursitis and patellar tendinopathy cannot be established with a single case report. Therefore, the authors recommend further well-designed studies to establish this association.

## Conclusions

Deep infrapatellar bursitis is a major cause of anterior knee pain, with trauma and overuse being the two most common causes. Due to the bursa lying deep into the patellar tendon, minor effusion may not become clinically evident. Chronic inflammation of DIPB may lead to patellar tendinopathy but this association needs further investigation. MSK-USG is a valuable tool that can effectively diagnose the condition relatively early and can also aid in the aspiration and injection of the bursa. Early diagnosis and management of this condition can potentially prevent chronic knee pain and improve function and quality of life.
